# A VMD-BP Model to Predict Laser Welding Keyhole-Induced Pore Defect in Al Butt–Lap Joint

**DOI:** 10.3390/ma17133270

**Published:** 2024-07-02

**Authors:** Wei Wang, Yang Dong, Fuyun Liu, Biao Yang, Xiaohui Han, Lianfeng Wei, Xiaoguo Song, Caiwang Tan

**Affiliations:** 1 State Key Laboratory of Precision Welding & Joining of Materials and Structures, Harbin Institute of Technology, Harbin 150001, China; wangwei_laser@163.com (W.W.); hanjiedongyang@163.com (Y.D.); hityangbiao@163.com (B.Y.); songxg@hitwh.edu.cn (X.S.); 2 Shandong Institute of Shipbuilding Technology, Weihai 264209, China; 3 CRCC Qingdao Sifang Co., Ltd., Qingdao 266111, China; 13793237339@139.com; 4 Nuclear Power Institute of China, State Key Laboratory of Advanced Nuclear Energy Technology, Chengdu 610213, China; wfenghit@163.com

**Keywords:** laser welding, keyhole-induced pore, VMD, BP neural network

## Abstract

The detection of keyhole-induced pore positions is a critical procedure for assessing laser welding quality. Considering the detection error due to pore migration and noise interference, this research proposes a regional prediction model based on the time–frequency-domain features of the laser plume. The original plume signal was separated into several signal segments to construct the morphological sequences. To suppress the mode mixing caused by environmental noise, variational modal decomposition (VMD) was utilized to process the signals. The time–frequency features extracted from the decomposed signals were acquired as the input of a backpropagation (BP) neural network to predict the pore locations. To reduce the prediction error caused by pore migration, the effect of the length of the signal segments on the prediction accuracy was investigated. The results show that the optimal signal segment length was 0.4 mm, with an accuracy of 97.77%. The 0.2 mm signal segments failed to eliminate the negative effects of pore migration. The signal segments over 0.4 mm resulted in prediction errors of small and dense pores. This work provides more guidance for optimizing the feature extraction of welding signals to improve the accuracy of welding defect identification.

## 1. Introduction

Aluminum alloy (6A01) is increasingly applied in Chinese High-Speed Railway (CHR) trains due to its properties of light weight, high specific strength, and corrosion resistance [1,2]. The roofs and side walls of trains are made of aluminum profiles and connected in the form of butt–lap joints. The laser welding of aluminum alloys has attracted particular attention because of its low heat input and large weld depth, with the ability to achieve deep penetration in butt–lap joints [3]. However, keyhole-induced pore defects easily form during the laser welding process, reducing the tensile strength of the joints and the safety of CHR trains [4,5]. Therefore, the detection of pore defects is a necessary procedure in train manufacturing. However, the post-welding inspection methods, such as X-ray detection, are time-consuming and expensive. It is necessary to investigate an efficient and reliable method to monitor keyhole-induced pore defects.

In recent years, the monitoring of keyhole-induced pores based on visual signals has attracted much attention. The signals have been collected through high-speed cameras, and different machine learning (ML) methods have been utilized to establish the relationships between the visual signals and keyhole-induced pores [6,7,8]. Luo et al. [8] established a radial basis function neural network model (RBFNN) to estimate the keyhole area in the laser welding process. They realized the detection of weld pores based on the abnormal fluctuation in the keyhole area. Zhang et al. [9] extracted the molten-pool features from a single image as the input of a convolutional neural network (CNN) to identify the keyhole-induced pores. The network had a 75% accuracy in predicting the porosity near the top surface but less accuracy in predicting deep porosity close to the bottom surface. An important reason for the low accuracy is that the migration of the porosity in the weld pool was not considered. Researchers have discovered that keyhole-induced pores migrate with the flow of the molten pool [5,10], which indicates that the locations of pore defects are different from where the pores are formed. Due to the migration of keyhole-induced pores, the method of feature extraction based on a single image only finds the positions of the pore formation rather than the pore defect location. Regarding the deep pores mentioned above, obvious pore migration results in large prediction errors. Therefore, it is necessary to consider pore migration when detecting keyhole-induced pores.

To consider the migration of pores, some researchers have proposed a regional prediction method based on image sequences. This method extracts features from continuous images within a small period and not a single image at a moment during the welding process. The prediction error caused by pore migration could be solved by selecting an image sequence that contains the information of both the pore formation and migration. Compared to a single image that only contains the time-domain features, the image sequence additionally includes the frequency-domain features. Ma et al. [11] confirmed that the frequency-domain features in a segment of signals had a significant effect on the accuracy of pore prediction. However, it is difficult to obtain the frequency-domain features from original plume signals because environmental interferences are always present in the welding process. Empirical model decomposition (EMD) and its implementation method were proposed as powerful tools to denoise the original welding signals [12]. Fan et al. [13] used the EMD method to process laser-MIG welding visual signals for welding state detection, revealing that EMD could promote reliable feature extraction of welding defect signals. Huang et al. [14] collected spectrum signals analyzed by EMD, and a support vector machine (SVM) model was proposed to predict the pores, with 90% accuracy. Although the reports mentioned above achieved good performances, it is a great challenge to obtain higher accuracy in the case of mode mixing in EMD. Mode mixing is defined as a single intrinsic mode function (IMF) containing both low-frequency and high-frequency oscillations of dramatically disparate scales [15,16]. Under this condition, the correlations among defects are lost when the frequency-domain features are extracted from the decomposed signals. Some researchers have proposed ensemble empirical mode decomposition (EEMD) to eliminate mode mixing by adding white noise [17]. However, mode mixing cannot be completely suppressed when the amplitude of white noise is small, and excessive white noise obscures the original signals, leading to partial loss of the features of defects [18]. A technique is needed to avoid interference with the original signals and to suppress mode mixing during signal processing.

Variational mode decomposition (VMD), an efficient signal processing method, can suppress mode mixing by adaptively matching the optimal center frequency of each mode and finite bandwidth based on the number of decomposed layers [19]. It is widely utilized for defect monitoring due to its accurate and high computing efficiency [20,21]. Kumar et al. [22] used VMD to decompose bearing vibration signals, which effectively enhanced the defect features and provided reference information for defect identification. Peng et al. [23] adopted the VMD method to decompose the electrical signals of MIG welding to eliminate noise and assess the weld quality. They trained a neural network and achieved 92.15% classification accuracy of weld defects. Similar to the signals mentioned above, the original plume morphological sequences were disordered, and it was necessary to denoise and enhance them. The VMD method shows great potential in suppressing mode mixing without extra interference, which increases the recognition of weld defects for feature extraction, such as keyhole-induced pores. However, the impact of the signal segment length on accuracy has not been considered in most studies on welding signal processing based on VMD.

The accuracies of the aforementioned research were lower than 93%. The backpropagation (BP) neural network is the most widely used artificial intelligence algorithm due to its flexible network architecture, nonlinear mapping capability, and generalization power compared to other networks [24]. Thus, the BP network was used in this study, and a better performance was exhibited, with 97.7% accuracy.

In this work, an VMD-BP model was proposed to detect the keyhole-induced pore defects in the Al butt–lap joint. The proposed model was used to detect the defects by collecting the laser plume signals with the temporal information. This VMD-BP model showed better performance compared with other models. The main contributions of the current work are as follows:

- The integration of VMD with BP neural networks enabled adaptive adjustment of the decomposition layers and demonstrated remarkable performance in signal decomposition and the prediction of porosity.
- The temporal information was considered to mitigate the effects of porosity migration, and the influence of signal segment length was evaluated.
- Widening the application of this model in the welding of high-speed trins, significantly improving manufacturing efficiency and ensuring the safety and reliability of high-speed trains.

## 2. Materials and Method

### 2.1. Materials and Reagents

The base materials are 6A01 aluminum alloy sheets with dimensions of 6 × 50 × 100 mm and 5454-H24 aluminum alloy sheets with dimensions of 3 × 40 × 100 mm. The 6A01 aluminum alloy sheet was milled off a region (3 mm × 5 mm × 100 mm), and the 5454 aluminum alloy sheet was overlapped on the milling surface and assembled in a butt–lap joint. The surfaces of utilized sheets were cleaned using acetone to discard the oil dirt, and polished by sandpapers to remove the oxide film.

### 2.2. Welding Procedures and Dataset Setting

**Figure 1:**
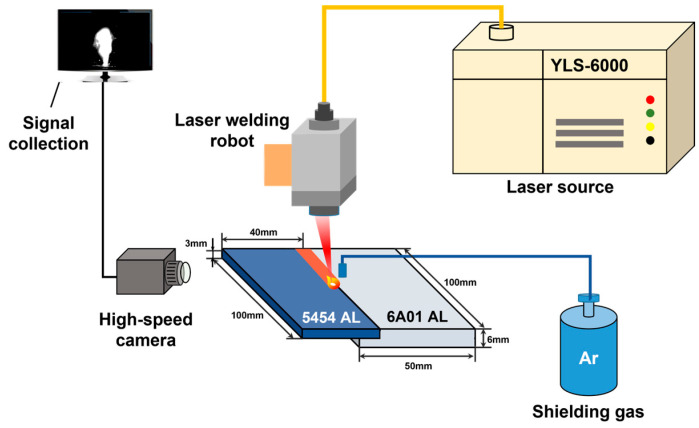
Schematic of laser welding process.

Figure 1 shows a schematic of welding process. The experimental setups were composed of an IPG YLS-6000 fiber laser (Thorlabs, Newton, NJ, USA), a KUKA robot, an industrial personal computer (IPC), and an IX I-SPEED 221 high-speed camera. The IPG YLS-6000 fiber laser system was used as heat source, which possessed a wavelength of 1070 ± 10 nm. The diameter of the laser beam was 300 μm and the laser power was set as 4200 W. For laser welding of aluminum alloy, the pore defects were unavoidable. However, it could be reduced to below 1% by choosing suitable processing parameters. In this study, the detection of porosity defects was focused. The KUKA robot was utilized to control the welding speed at 1.2 m/min. The pure argon was used as shielding gas with a flow rate of 10 L/min. The angle between the laser axis and the vertical direction was set as 10° to prevent possible optical component damage. The high-speed camera was used to observe the morphology of the laser plume. The light filter was placed in front of the lens to suppress the light interference and obtain high-contrast images. The frame rate was set to 2500 fps, and the image resolution was 544 × 948 pixels. The camera was connected to the IPC and fed the images to the IPC.

The image set totally consisted of 120,000 images. A single data point was formed by every consecutive set of 10 images. Each data point was annotated with a label (0 represented no porosity, 1 represented porosity) to indicate the presence or absence of porosity. Consequently, each weld seam, comprising 5000 images, was condensed into 500 data points, enabling a comprehensive assessment of the porosity condition of the entire weld seam. Hence, the total dataset contained 12,000 data points composed of 3654 data points for porosity and 8346 data points for no porosity. The dataset was divided into two different units; 8400 samples were selected in the training set and 3600 samples were chosen for the test set, with a ratio of 7:3. Each data point was fed to the model for preprocessing and calculation. The weld seam represented by each data point was judged for porosity. The model was performed on PyEMD in the python 3.11 programming environment, and the VDM was directly invoked by PyEMD. The alpha was set to 2000 to ensure each IMF had an appropriate bandwidth. The initial k was set within a range of 3 to 10 to iteratively determine the optimal number of decomposition layers through an adaptive approach. The tol was set with 10^−7^ to obtain excellent performance, and the init was set to 1.

### 2.3. Data Preprocessing

#### 2.3.1. Image Process

**Figure 2:**
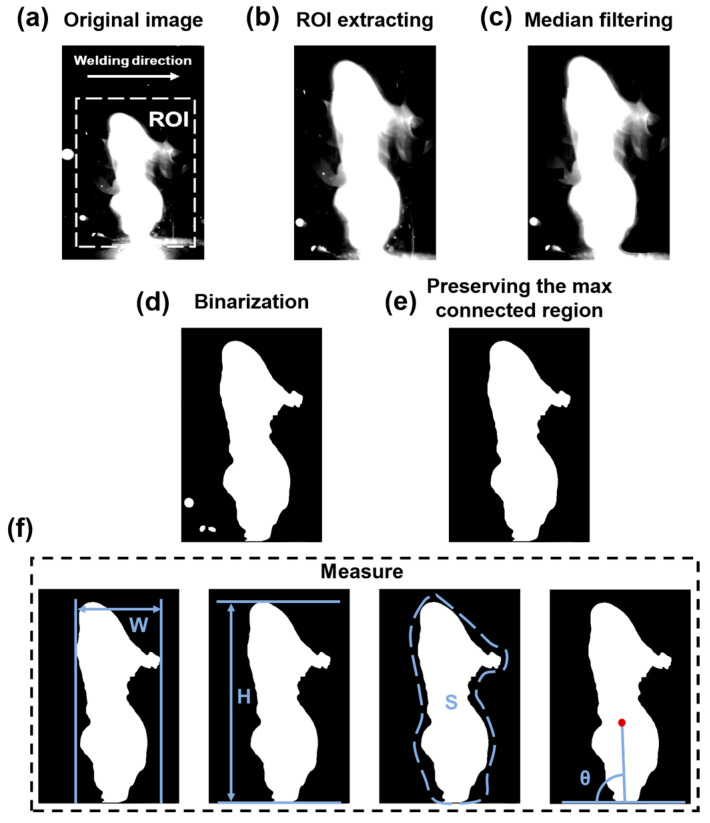
Image processing scheme: (**a**) original image; (**b**) ROI extracting; (**c**) median filtering; (**d**) binarization; (**e**) the max connected region; (**f**) measurement of width, height, area, and angle.

This work extracted the plume morphological features in the welding process, including the width, height, area, and angle. The image procedure is displayed in Figure 2. The first step was to select and crop the original image regions of interest (ROI), as shown in Figure 2a,b. Figure 2c shows the median filtering method that was utilized to remove noise in the images. In addition, the Otsu threshold method [25] was used in ROI to realize threshold segmentation in Figure 2d. After that, the maximum connect region was regarded as the morphology of the plume, as shown in Figure 2e. Finally, the features of the laser plume were quantified, including the width W (the difference in horizontal coordinate between the right- and leftmost point in connected domain), the height of plume H (the difference between the highest and lowest pixels), the area S (the total area of pixels with a gray level of 255, the total number of points in connected domain), and the inclination angle θ (the inclination degree between the horizontal line and the line formed by the centroid of connected domain and the middle point of bottom).

#### 2.3.2. Signal Decomposition

**Figure 3:**
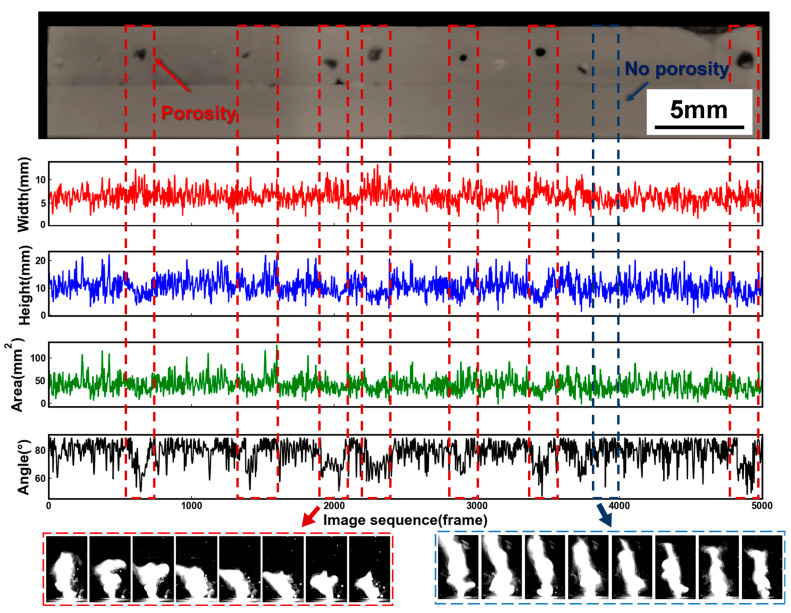
Signal diagnosing the porosity defect.

To correspond with the positions of the pores, it was necessary to arrange the plume images in chronological order and then extract the morphological signals, as described in Section 2.3.1. A total of 24 weld seams were obtained during the experiment, and 5000 plume images were collected in each weld seam. Figure 3 reflects the variation in laser plume morphology in 5000 images, representing for one weld seam. Upon the occurrence of keyhole-induced pores, the morphological signals fluctuated violently in a short segment. Specifically, the level of plume width increased, while that of the plume height, area, and angle decreased. The normal morphological signals of the laser plume slightly fluctuated due to the inevitable environmental noise, which had a negative impact on the recognition of pores. Therefore, the signals required decomposition to separate the defect signals from the original non-stationary plume signals.

The VMD algorithm is a non-recursive and adaptive method, which we used to decompose the signals to reduce data fluctuation and suppress noise [26]. The basic process was to search for the optimal variational model by iteration. In the decomposition process, the function and center frequency of each mode were updated continuously. The decomposition layer K was a key parameter that affected the performance of VMD, which was preset before decomposition. The decomposition procedures were as follows:

Firstly, the Hilbert transformation [27] was utilized to obtain the marginal spectrum for each mode signal. Secondly, the exponential terms were added to each mode signal’s estimated center frequency, and the spectrum was modulated to the baseband. The VMD method can be described as a constrained variational problem, as shown in Equation (1):

(1) $$\begin{array}{l} \underset{\left\{\left.u_{k}\right\}\right.,\left\{\left.\omega_{k}\right\}\right.}{min}\left\{{\sum_{k=1}^{k}\left\|\partial_{t}\left[\left(\delta \left(t\right)+\frac{j}{\pi t}\right)*u_{k}\left(t\right)\right]e^{-j\omega_{k}t}\right\|}_{2}^{2}\right\}\\ s.t.\sum_{k=1}^{k}u_{k}=s \end{array}$$

where *∂_t_* represented the gradient operation, * was the convolution operation, *δ*(*t*) was the Dirac distribution, *µ_k_*(*t*) was the set of kth decomposed mode, *ω_k_*(*t*) represented the center frequency of each mode in *µ_k_*(*t*), *k* was the number of components into which the signal was decomposed, *j* was the imaginary part, and *s* reflected the original signals.

Thirdly, to for search the optimal variational constraint mode solution, the Lagrange multiplier and quadratic penalty factor were used. The augmented Lagrange function [28] was obtained, as shown in Equation (2):

(2) $$\begin{array}{l} L\left(\left\{\left.u_{k}\right\}\right.,\left\{\left.\omega_{k}\right\},\lambda \right.\right)=\alpha {\sum_{k}\left\|\partial_{t}\left[\left(\delta \left(t\right)+\frac{j}{\pi t}\right)*u_{k}\left(t\right)\right]e^{-j\omega_{k}t}\right\|}_{2}^{2}\\ +{\left\|f\left(t\right)-\sum_{k}u_{k}\left(t\right)\right\|}_{2}^{2}+\langle \lambda \left(t\right),f\left(t\right)-\sum_{k}u_{k}\left(t\right)\rangle \end{array}$$

where *ω_k_* reflected the *k*-th center frequency of mode, and *λ* was the Lagrange multiplier.

Fourthly, the alternating multiplicative operators were utilized to obtain the optimal solution, the decomposition layers K and composition IMFs. The specific decomposition procedures of the VMD were divided into initialization, as in Equation (3), updating *u_k_* and *ω_k_* as in Equations (4) and (5), updating *λ* as in Equation (6), and achieving decomposition layers K and composition IMFs.

(3) $${\overset{⌢}{\omega }}_{k}^{1}={\overset{⌢}{u}}_{k}^{1}={\overset{⌢}{\lambda }}_{k}^{1}=n=0$$

(4) $${\overset{⌢}{u}}_{k}^{n+1}(\omega )=\frac{\overset{⌢}{S}(\omega )-\sum_{i<k}{\overset{⌢}{u}}_{i}^{n+1}(\omega )+\sum_{i>k}{\overset{⌢}{u}}_{i}^{n}(\omega )+\frac{\overset{⌢}{\lambda }(\omega )}{2}}{1+2\alpha (\omega -\omega_{k})}$$

(5) $$\omega_{k}^{n+1}=\frac{\int_{0}^{\infty }\omega {\left|{\overset{⌢}{u}}_{k}^{n+1}(\omega )\right|}^{2}d\omega }{\int_{0}^{\infty }{\left|{\overset{⌢}{u}}_{k}^{n+1}(\omega )\right|}^{2}d\omega }$$

(6) $${\overset{⌢}{\lambda }}^{n+1}={\overset{⌢}{\lambda }}^{n}(\omega )+\tau (\overset{⌢}{s}(\omega )-\sum_{k}u_{k}^{n+1}(\omega ))$$

Finally, the termination condition was defined as the absolute mean square of the IMFs obtained from two consecutive iterations being less than 10^−7^. With an increase in iterations, VMD stopped proceeding when the termination condition was satisfied. The decomposed signals contained a series of IMFs with different frequencies. The spectral distribution could be acquired via Fourier transform.

#### 2.3.3. Feature Extraction

**Figure 4:**
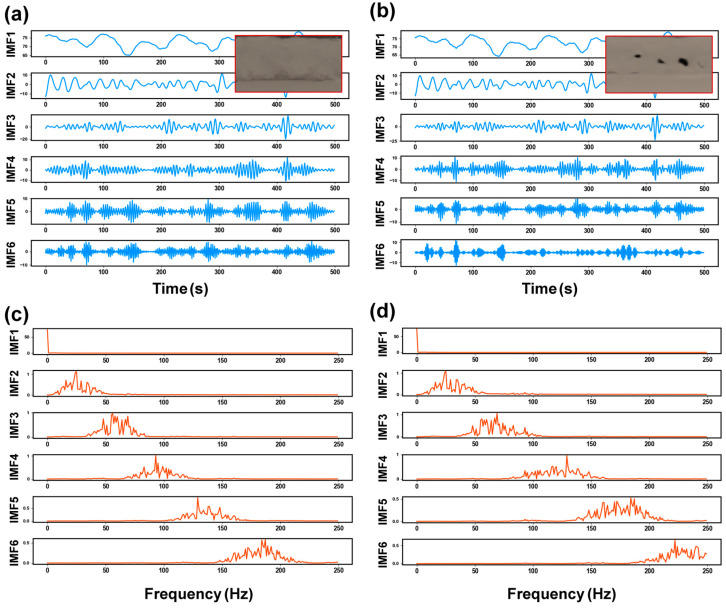
Decomposition results of plume angle signal: time-domain distribution: (**a**) no pores; (**b**) pores; frequency spectrum: (**c**) no pores; (**d**) pores.

Figure 4 shows the decomposition results of the laser plume angle signals based on VMD. The IMFs containing different frequencies were clearly separated after decomposition by VMD. The decomposition frequencies of signals from weld segments with and without porosity gradually increased with the decomposition layers. When pores occurred in the weld seam, the component frequencies in Figure 4b increased more rapidly.

Compared to the time-domain or frequency-domain features individually, time–frequency-domain features are regarded as a detailed and accurate method to describe the morphology variation in laser plume. Each IMF discomposed by VMD is classified as a source of time-domain features or frequency-domain features. The different kinds of features were extracted and combined to form a dataset of time–frequency-domain features. The Fourier transform was adopted to obtain the frequency distribution of IMF quantitatively. Figure 4c,d shows the frequency spectrum of the decomposed signals after the Fourier transform. The IMFs lower than 50 Hz were represented as the low-frequency components, including IMF1 and IMF2. The remaining modes from IMF3 to IMF6 were treated as high-frequency components. The frequencies corresponding to the maximum amplitude were collected as the frequency-domain features. Figure 4c shows the state without keyhole-induced pores. The center frequency of the high-frequency component increased from 54 Hz to 182 Hz. When the keyhole-induced pore appeared, the center frequency of the high-frequency component increased from 75 Hz to 229 Hz, as shown in Figure 4d. As described in Section 2.3.2, the appearance of keyhole-induced pores led to violent fluctuations, suggesting more high-frequency components existed in the decomposed results. Therefore, the center frequencies of IMF3 to IMF6 were utilized as the frequency-domain feature of the plume morphology signals to predict the KI pore formation.

Sample entropy (SampEn) is an index used to quantify the complexity of time series, unhampered by signal length and adept at capturing the intricacy and uncertainty embedded within time-series signals, as exhibited in Equation (7):

(7) $$SampEn(m,r,N)=-\log\left(\frac{\sum_{i=1}^{N-m+1}C_{i}}{\sum_{i=1}^{N-m}B_{i}}\right)$$

where *m* reflected the length of vector, *r* was the tolerance for defining similarity, *N* represented the total length of the time series, *C_i_* counted the number of template matches for *i*, and *B_i_* was the similar matches for the *i* to provide a normalization factor.

**Figure 5:**
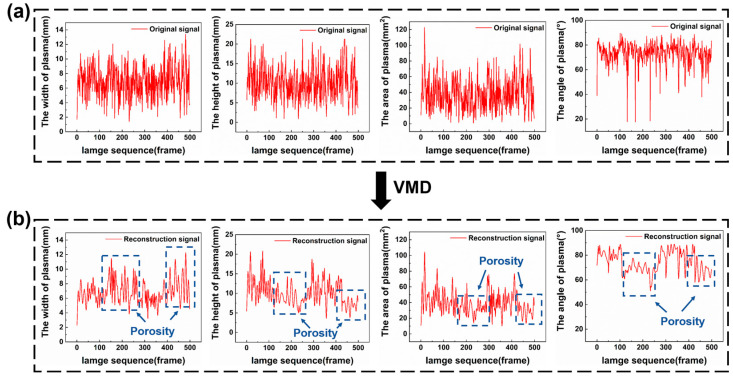
Signal reconstruction by VMD: (**a**) original signals; (**b**) reconstructed signals.

The time-domain features were extracted based on the low-frequency component. The plume morphology signals were restructured with low-frequency components to reduce the noise and improve feature distinction. The SamEn of each component is shown in Table 1. Because only similar values of SampEn with the row data displayed the same fluctuation trend, the IMF1 and IMF2 components were chosen for the reconstruction. Figure 5 displays the reconstruction signals of four morphology signals reconstructed by the VMD low-frequency components IMF1 and IMF2. Significant differences were found in the average level and variance of restructured signals corresponding to the pores’ formation. The average level of plume width was higher, while that of plume height, area, and angle was lower with the porosity defect compared to no-porosity defect. In addition, when porosity defects appeared, the variances represented for the fluctuation degree plume morphology features were higher compared with the signals without a porosity defect. Thus, the average and variance of reconstructed signals were extracted as the time-domain features to recognize the keyhole-induced pores.

**Figure 6:**
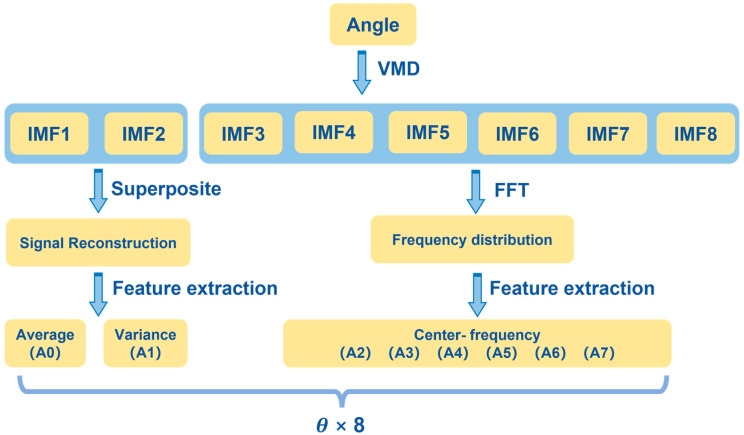
Feature extraction process for angle of laser beam.

In summary, the set of the keyhole-induced pores features included both time-domain features and frequency-domain features. The frequency-domain features were the center frequencies of high-frequency components, and the time-domain features were the average and the variance of signals reconstructed by low-frequency components. The feature extraction method for angles is shown in Figure 6. The two low-frequency components were utilized to reconstruct the signals, and then the average and variance were extracted as time-domain features. The center frequencies of the remaining components were the six frequency-domain features. Therefore, the plume angle was extracted as eight features in total. Similar feature extraction was utilized for all morphological signals, and a total of twenty-six features were extracted in each signal sequence.

#### 2.3.4. Division of Signal Segments

**Figure 7:**
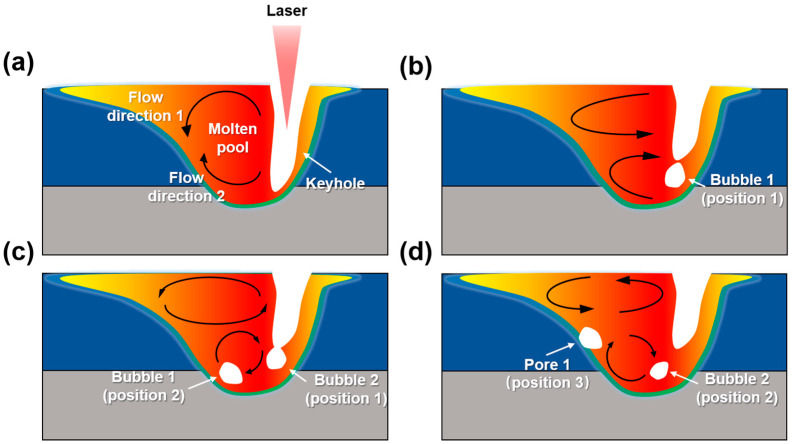
The procedure of KI pore formation and migration: (**a**) normal state; (**b**) bubble formation; (**c**) bubble migration; (**d**) KI pore transformation.

Figure 7 shows the representative processes of the keyhole-induced pore formation and migration [10,29]. During the laser welding, the location of the keyhole absorbed the most laser beam energy, and the metal vapored from there. Thus, the keyhole was a cavity filled with metal vaporization. The keyhole was opened continually during the normal welding process, as shown in Figure 7a. Once the keyhole was closed abnormally, the metal vapor was trapped in the molten pool and bubble 1 was formatted, as shown in Figure 7b. In addition, two vortexes in the contrary directions were formatted at the molten pool surface and bottom because of the molten metal flow, as shown in Figure 7a. With the violent clockwise vortex at the molten pool bottom, bubble 1 overcame the obstacle of viscosity force and surface tension gradient and migrated from position 1 in Figure 7b to position 2 in Figure 7c and position 3 in Figure 7d. Due to the continuing solidification and movement of the solidification front, bubble 1 was trapped in position 3 and transformed into a keyhole-induced pore. In Figure 7c,d, the other bubble, bubble 2, was formed via a similar migration process. Therefore, the location of the keyhole-induced pore was different from the bubble’s initial formation position [30].

To reduce the prediction errors due to the pore migration, the region prediction method was used in this research. The region prediction model was achieved by a sliding window. Each weld seam was scanned by the sliding window and divided into several signal segments. The VMD method was utilized to decompose the signal segments collected in the region covered by the sliding window. The experiments showed that with the minimum pore size, the plume variation range was about 25 frames, which was about 0.2 mm. Therefore, the signal segment lengths chosen for the area prediction were multiples of 0.2 mm, which were 0.2 mm, 0.4 mm, 0.6 mm, and 0.8 mm, corresponding to 25 frames, 50 frames, 75 frames, and 100 frames.

### 2.4. Calculations and Modelling

#### 2.4.1. Architecture of BP Neural Network

**Figure 8:**
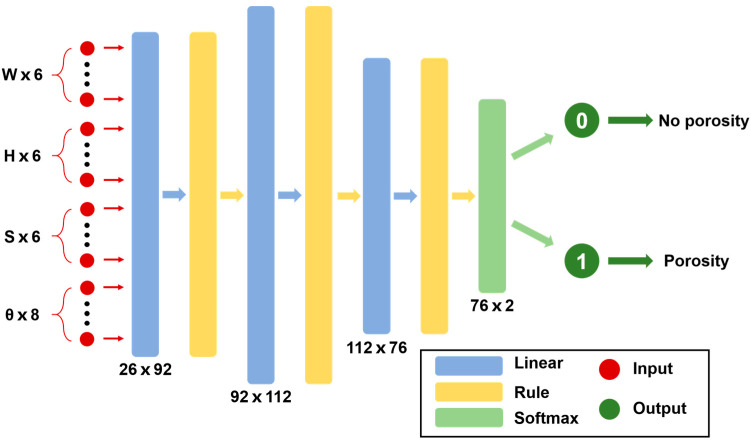
The construction of BP-Net.

The prediction model was established by a BP neural network in this work. The generalization ability of BP neural network was strong, which meant the BP neural network was suitable to solve nonlinear prediction problem [31,32]. A three-layer neural network was established, as shown in Figure 8. The numbers of neurons in each hidden layer were 92, 112, and 76, respectively, which were proven to be the optimal network construction by repeated debugging. A total twenty-six features extracted by each signal segment were utilized as inputs. The output was set to 0 or 1 according to the actual weld picture; 0 represented no-porosity state, and 1 represented the porosity state.

The BP neural network operated through the following procedures:

Firstly, the neural network was initialized.

Secondly, according to the input vector X, the output of hidden layer H was calculated. The calculation process is shown in Equation (8):

(8) $$H_{j}=f(\sum_{i=1}^{n}\omega_{ij}x_{i}-a_{j}),j=1,2,\ldots \ldots ,l$$

where *l* reflected the number of neuron hidden layers, *n* was the input number of layer nodes, *f* represented the activation function of each hidden layer (the ReLU function [33] was utilized as activation function in this work), *ω_ij_* reflected the connection weight between adjoining layer, and *a_j_* was the *j*th hidden layer threshold.

Thirdly, the previous hidden layer output vector was regarded as the input vector for next hidden layer, and the next hidden layer output vector was calculated through Equation (7). When all the hidden layer output vectors were calculated, the hidden layer operation was finished.

Fourthly, the last output vector was inputted to the softmax layer to calculate the affiliation probability for porosity category or no-porosity category. The output 0 or 1 was then decided.

Fifthly, the error e between prediction output and truth label was calculated. If the error was larger than the regulation limit, the connection weights and thresholds were updated through feedforward optimization until the error was lower than the regulation limit. The optimization process is displayed as Equations (8) and (10).

(9) $$\omega_{ij}=\omega_{ij}+\eta H_{j}(1-H_{j})x(i)\sum_{k=1}\omega_{jk}O_{m},i=1,2,\ldots \ldots ,n;j=1,2,\ldots \ldots ,l$$

(10) $$a_{j}=a_{j}+\eta H_{j}(1-H_{j})\sum_{k=1}\omega_{jk}O_{m},j=1,2,\ldots \ldots ,l$$

where *η* was the learning rate.

#### 2.4.2. Correlation Analysis of BP Neural Network

For the model, the Spearman’s rank coefficient of correlation was used to gauge the association between features and the label, as shown in Equation (11):

(11) $$\rho =\frac{\frac{1}{n}\sum_{i=1}^{n}\left(\left(R\left(x_{i}\right)-\bar{R\left(x\right)}\right)\cdot \left(R\left(y_{i}\right)-\bar{R\left(y\right)}\right)\right)}{\sqrt{\left(\frac{1}{n}\sum_{i=1}^{n}{\left(R\left(x_{i}\right)-\bar{R\left(x\right)}\right)}^{2}\right)\cdot \left(\frac{1}{n}\sum_{i=1}^{n}{\left(R\left(y_{i}\right)-\bar{R\left(y\right)}\right)}^{2}\right)}}$$

where *R(x)* and *R(y)* denoted the rank orders of variables *x* and *y*, respectively. $\bar{R\left(x\right)}$ and $\bar{R\left(y\right)}$ were mean ranks of *x* and *y*, *x* meant the extracted features, *y* meant the label.

## 3. Results and Discussion

### 3.1. The Performance of Different Decomposition Methods

**Figure 9:**
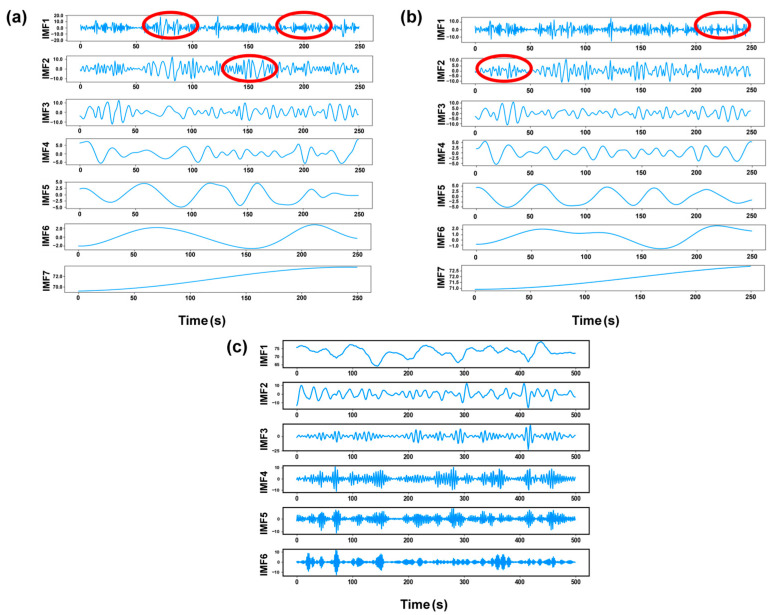
The time-domain waveforms of IMFs: (**a**) EMD; (**b**) EEMD; (**c**) VMD.

**Figure 10:**
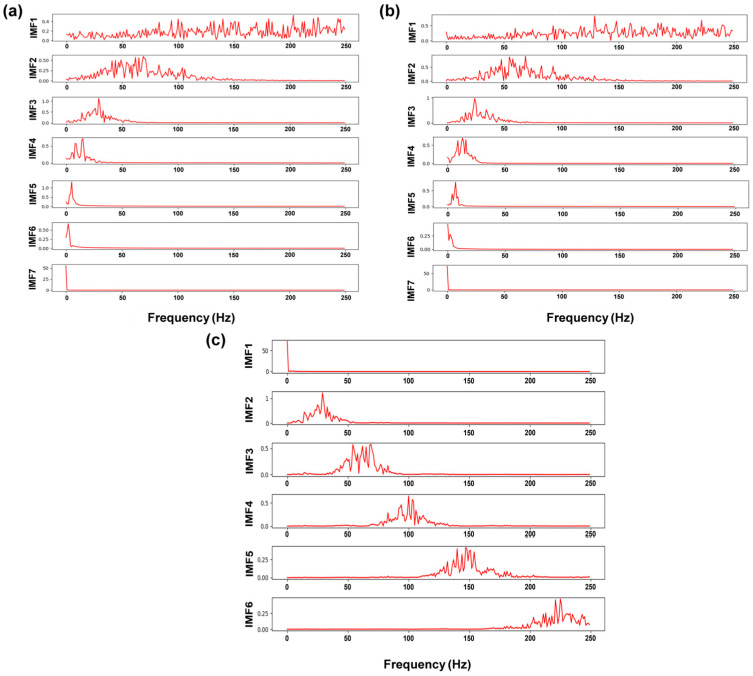
The frequency spectra of IMFs: (**a**) EMD; (**b**) EEMD; (**c**) VMD.

To evaluate the performance of VMD on the original morphology signals, the same signal sequence of the plume angle was decomposed by EMD, EEMD, and VMD to obtain the IMFs. The Fourier transform was utilized to process the IMFs to acquire the frequency distribution of the signals. Figure 9 shows the time-domain waveforms of decomposed IMFs, and Figure 10 exhibits the corresponding frequency spectra. The original signals were decomposed into seven IMFs after being processed by the EMD and EEMD. The previous section illustrated that the optimal number of decomposition layers for the VMD method was six. Both EEMD and EMD were unable to modify the numbers of IMFs. Because of the large number of extremity points caused by the unstable fluctuation, the number of IMFs obtained from EEMD and EMD decomposition was also higher than that in VMD decomposition. Excessive numbers of decomposition layers led to redundant feature extraction and complex datasets. In addition, it was found that the EMD and EEMD methods had partial position mixing between IMF1 and IMF2 (labeled by a red line). With the application of VMD in the signal decomposition, the mode mixing was difficult to observe. The spectral analysis of the signals also confirmed that the high-frequency components of EMD and EEMD were not clearly decomposed. However, the frequencies of the components in the VMD decomposed signals were basically distributed in different intervals, and the mode mixing problem was almost sufficiently suppressed.

To specifically compare the degree of mode mixing between the components, the signal was calculated using the orthogonality index (IO) proposed by Huang et al. [34], as shown in Equation (12).

(12) $$IO=\sum_{t=0}^{T}(\sum_{i=1}^{n}\sum_{j=1}^{n}imf_{i}\left(t\right)\times imf_{j}\left(t\right)/x{\left(t\right)}^{2})$$

where *x*(*t*) was the original plume signal, *imf_i_*(*t*) was the IMF component, and the residual denoted the *imf_n_*(*t*). Obviously, when there was no mixing between IMFs, the *IO* was zero. As the signal mixing level increased, the *IO* value became larger. The *IO*s of the three methods for the IMFs were 3.247, 1.591, and 0.107, indicating that the VMD decomposition effectively reduced the mode mixing phenomenon of the plume signals. This was mainly related to the decomposition mechanism of VMD.

With the appearance of the KI pore in laser welding, the original plume signals showed a transient and violent fluctuation. EMD and EEMD were unable to control the signal bandwidth, and they were prone to mode mixing when processing the nonlinear and non-smooth signals [35]. The appearance of mode mixing showed that the signal decomposition was incomplete, which made the center frequency lose the significance of representing the frequency-domain feature of the decomposed signals. VMD used the principle of controlling the bandwidth of the decomposed signals and iterated the decomposed signals in different frequency domains to effectively suppress the mode mixing [19,36]. Due to the powerful denoising ability of VMD, the defect features extracted after decomposition were more easily identified, which was beneficial in improving the robustness of the prediction model.

### 3.2. The Performance of Neural Network Prediction

**Figure 11:**
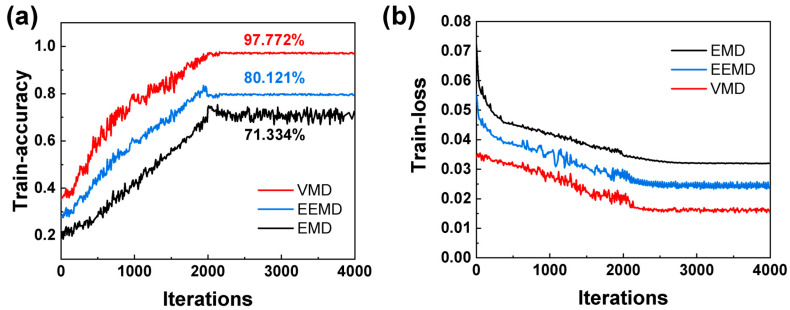
The training process of BP neural network: (**a**) the accuracy; (**b**) the loss curve.

A regional prediction method with a signal segment length of 0.4 mm was selected as an example to demonstrate the training process. Figure 11 reflects the variation in accuracy and loss of the prediction model during the training process. The training process was divided into 20 epochs, with 200 iterations in each epoch and 4000 iterations. The accuracy variation in the training process is described in Figure 11a. For VMD, the accuracy increased rapidly from 35% to 83%, with iterations from 0 to 1500. The accuracy of the prediction model maintained a slight fluctuation after 2000 iterations. An accuracy of 97.77% was the maximum, with 2100 iterations in all training epochs. The accuracies of EEMD and EMD were 80.12% and 71.334%, respectively, which were much lower than VMD. To analyze the training process, the loss curve is displayed in Figure 11b. For VMD, the fast decreasing trendy from 0 iterations to 1500 indicated that the prediction model was optimized gradually. With 1500 to 2000 iterations, the violent fluctuating loss value indicated that the prediction process was trapped in a locally optimal solution. With 2000 iterations, the loss value decreased again, showing that the prediction model had escaped from the saddle point and was optimized again. When the iterations were increased to 2100, the loss value fluctuated in a small range, indicating that the optimal results were obtained. For EEMD and EMD, the trends of loss were similar to VMD. However, the loss of EEMD and EMD converged to 0.2349 and 0.03201, respectively.

**Figure 12:**
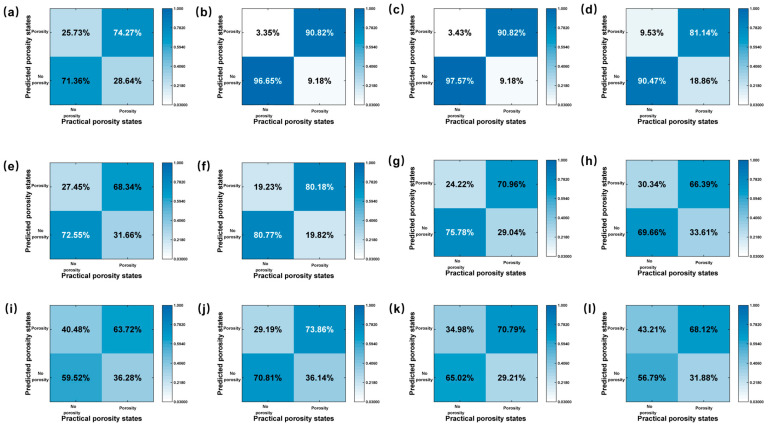
Confusion matrix of regional prediction for different model. VMD: (**a**) 25 frames; (**b**) 50 frames; (**c**) 75 frames; (**d**) 100 frames; EEMD: (**e**) 25 frames; (**f**) 50 frames; (**g**) 75 frames; (**h**) 100 frames; EMD: (**i**) 25 frames; (**j**) 50 frames; (**k**) 75 frames; (**l**) 100 frames.

To verify the validity of the prediction model, an experiment was designed with the same welding parameters and conditions. The same feature extraction method as the training set was used to obtain laser plume features, and the network was used to predict weld porosity. To investigate the effect of different signal segment lengths on the prediction results, confusion matrixes were calculated, as shown in Figure 12. For VMD, the confusion matrixes are shown in Figure 12a–d. For EEMD, the confusion matrixes are shown in Figure 12e–h. For EMD, the confusion matrixes are shown in Figure 12i–l. Figure 12a showed a result of 0.2 mm signal segment, and the accuracy of prediction for pores was only 74.27%. The accuracy of the neural network was significantly improved by expanding the length of the signal segment. With a signal segment length of 0.4 mm, as in Fig. 12b, the porosity prediction accuracy was 90.82%. When the signal segment length was increased from 0.4 mm to 0.8 mm, the prediction accuracy for pores decreased from 90.82% to 81.14%. This indicated that the signal segment length was the key parameter affecting the accuracy. A better value of this parameter was 0.4 mm, containing 50 frames. For EEMD and EMD, it has been observed that the trends exhibited by varying the lengths of signal segments when generating confusion matrixes for both the EMD and EEMD showed a remarkable resemblance to that seen with VMD. Specifically, these analyses consistently indicated that a signal length of approximately 0.4 mm had a better performance. The mechanism of the effect of this parameter on the network accuracy is discussed in the following sections.

### 3.3. Influence Mechanism of Signal Segment Length

**Figure 13:**
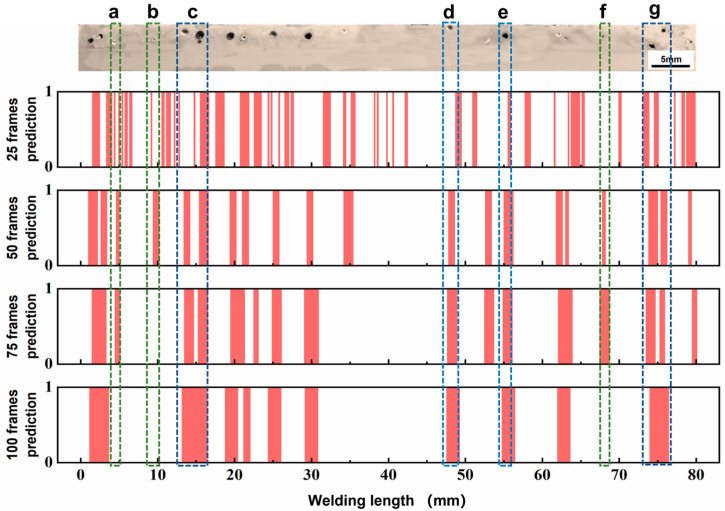
Prediction results of different frames signal segment (25, 50, 75, 100). a, b, f: the pores less than 0.3 mm in diameter; c, g: several pores at short distances; d, e: the pores larger than 0.3 mm in diameter.

Figure 13 shows the prediction results for the same weld seam scanned by different signal segments. For the 0.2 mm signal segment, the results revealed that there were two main reasons for the low accuracy. Firstly, many regions labeled as no pores were judged as having pores existing in the prediction results. This was attributed to the transient fluctuation caused by the external environmental interference such as the shielding gas flow. The fluctuation made the laser plume show similar variations in morphological features for the formation of keyhole-induced pores. It was found that the instantaneous fluctuation phenomenon lasted for 5–10 frames and had a length of 0.08 mm–0.1 mm. Because the length of the signal segment was only 0.2 mm, this fluctuation could not be ignored, which caused the position without pores to be identified as the pore location. The other type of prediction error was the location problem, where pores were predicted as no pores. By comparing the images, a significant hysteresis in the predicted locations corresponded to the actual pores, such as d and e in Figure 13. However, this phenomenon almost disappeared when the length of the signal segment was expanded to 0.4 mm. This was because the 0.2 mm signal segment was too short to cover the range of pore formation for localization. The negative effect of pore migration on the prediction accuracy still existed. In addition, it was difficult to decompose by VMD because the signals contained few data, and the fluctuation was not regular. A reasonable signal segment length was useful to reduce the interference of laser plume transient mutation and pore migration on the prediction results.

The result in Section 3.2 revealed that the optimal signal segment length was 0.4 mm, which contained 50 frames. When the length of the signal segment expanded from 50 frames to 100 frames, the accuracy of the neural network decreased. A significant difference for different signal segments occurred in predicting pores of different sizes. The prediction results based on different signal segments were correct for large pores (>0.3 mm in diameter). For pores less than 0.3 mm in diameter, such as positions a, b, and f in Figure 13, a signal segment with 50 frames could accurately find the pore location; however, a signal segment which contained 75 frames ignored some of them. When the signal segment length increased to 100 frames, the neural network was unable to recognize the small pores. In addition, unstable keyhole flow during welding induced the appearance of dense porosity at short distances, as shown at c and g in Figure 13. For the pores larger than 0.3 mm in diameter, such as positions d and e in Figure 13, a signal segment with 50, 75, and 100 frames could accurately find the pore location. The dense pores were separated precisely by the 50-frame signal segment. The prediction results of both 75- and 100-frame signal segments showed the presence of porosity in several continuous regions, which made the assessment of the weld quality difficult. Therefore, the regional prediction method based on a signal segment of 50 frames has advantages in the identification of small pores and the separation for dense pores, which led to optimal accuracy in pore prediction.

**Figure 14:**
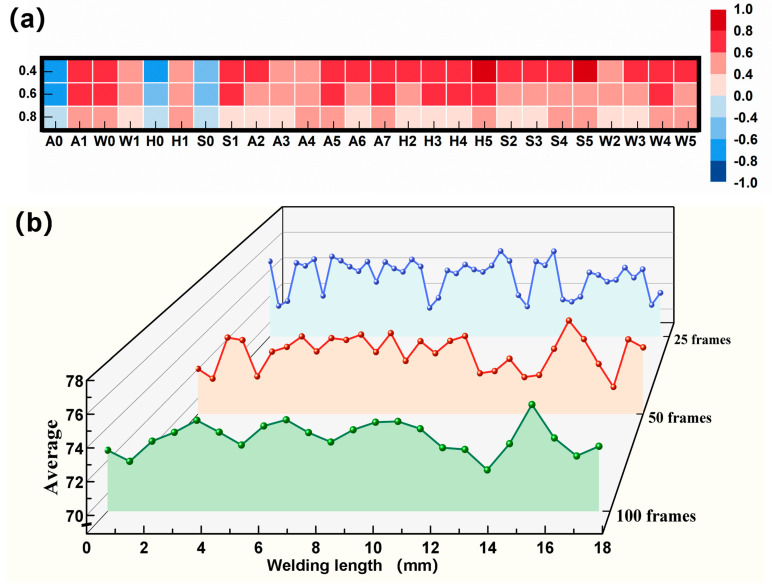
Correlation analysis of features: (**a**) correlation coefficients for total features; (**b**) A0 feature.

A weld seam from the verification set was intercepted from 1.5 mm to 21.5 mm for analysis, as shown in Figure 14. The weld seam contained the two types of porosity that caused the prediction deviations mentioned above. Considering the quality of the extracted features at different window lengths, the Spearman correlation coefficient was utilized to calculate the correlation between the different features and the label sequences. Figure 14a shows the distribution of the correlation coefficients, where all significance factors were less than 0.05. The vertical axis was the signal segment length, and the horizontal arrangement was the 26 features extracted from plume morphology, in which A meant the 8 extracted features formed an angle, W meant the 6 extracted features formed width, H meant the 6 extracted features formed height, and S meant the 6 extracted features formed area. It was found that the correlation between features and labels decreased as the window length increased. This situation was mainly caused by the dilution of features.

The effect of feature dilution on the feature correlation decline was explained using the distribution of the A0 feature as an example in Figure 14b. There was an obvious drop corresponding to the pore appearance when the weld seam was scanned by a 0.4 mm signal segment. As the signal segment length increased, the variation in A0 features was not obvious. A similar situation existed in the rest of the features. Excessively expanding the signal segment length was the key factor leading to feature dilution. This was because the scale of the feature sequence fluctuations corresponding to the small pores was neglected compared to the whole signal segment. Feature sequences containing pores were diluted by a large number of signals representing no pores, which contributed to the smooth fluctuation of the A0 feature. For the dense pores, the A0 feature was at a sustained low level, and the morphological variation in the laser plume between pores was ignored. Therefore, the feature dilution caused by the large signal segment obscured the correspondence between features and labels, which deteriorated the predictive performance of the neural network.

## 4. Conclusions

In this research, we proposed a prediction model for porosity defects based on VMD-BP, which considered the influence of pore migration. The feasibility of assessing the porosity defect states by monitoring the laser plume morphological changes was demonstrated. The conclusions are as follows:

(1) The VMD method was newly utilized to process the morphological signals of the laser plume in this study. More high-frequency components existed in the decomposition results, corresponding to the presence of keyhole-induced pores. By comparing with the EMD and EEMD methods, VMD had better robustness in processing the nonlinear and non-smooth welding signal. The decomposed signals had lower orthogonal coefficients, indicating that the mode mixing was sufficiently suppressed.
(2) The time–frequency-domain feature extraction method is based on the signals after being decomposed by VMD. The feature dataset consisted of frequency-domain features from high-frequency components and time-domain features from low-frequency components. A BP neural network was developed and achieved 97.77% accuracy in pore prediction.
(3) The length of the signal segment was a key parameter affecting the BP neural network accuracy. The best signal segment length was 0.4 mm. The reduction in the signal segment length failed to escape the prediction errors due to pore migration. Excessively long signal segments ignored the position of small-sized pores and the distinguishing dense pores, which was attributed to the decrease in correlation between features and defects caused by the feature dilution.

## Figures and Tables

**Table 1:** Sample entropy for each modal component.

Components	SampEn
Raw data	0.358
IMF1	0.357
IMF2	0.362
IMF3	0.652
IMF4	0.542
IMF5	0.674
IMF6	0.598

## Data Availability

The raw data supporting the conclusions of this article will be made available by the authors on request.
